# TRIM21 promotes type I interferon by inhibiting the autophagic degradation of STING via p62/SQSTM1 ubiquitination in systemic lupus erythematosus: TRIM21 promotes IFN-I by inhibiting STING autophagy via p62 in SLE

**DOI:** 10.3724/abbs.2025046

**Published:** 2025-03-31

**Authors:** Chen Li, Ang Ma, Yu Bai, Zitao Liu, Linghan Tian, Ziyuan Wang, Huaishun Ma, Zhengpu Chen, Zhengheng Gao, Shijie Feng, Ping Fu

**Affiliations:** 1 Department of Rheumatology and Clinical Immunology the Second Affiliated Hospital of Kunming Medical University Kunming 650032 China; 2 Department of Urology Yunnan Cancer Hospital the Third Affiliated Hospital of Kunming Medical University Peking University Cancer Hospital Yunnan Kunming 650118 China; 3 Department of Scientific Research Yunnan Cancer Hospital the Third Affiliated Hospital of Kunming Medical University Peking University Cancer Hospital Yunnan Kunming 650118 China; 4 Cancer Institute Yunnan Cancer Hospital the Third Affiliated Hospital of Kunming Medical University Peking University Cancer Hospital Kunming 650118 China; 5 Department of Health Management and Tumor Screening Center Yunnan Cancer Hospital the Third Affiliated Hospital of Kunming Medical University Peking University Cancer Hospital Yunnan Kunming 650118 China

**Keywords:** systemic lupus erythematosus, cGAS-STING signaling, autophagy, ubiquitylation, TRIM21, p62/SQSTM1

## Abstract

The cGAS-STING signaling pathway serves as a pivotal surveillance mechanism for cytosolic double-stranded DNA (dsDNA) detection in mammalian systems. While STING-mediated type I interferon production is crucial for host defense, sustained activation of this pathway contributes to autoimmune pathologies, including systemic lupus erythematosus (SLE). Maintaining immune homeostasis requires precise regulation of STING activity to prevent hyperactivation. Our study identifies TRIM21 as a novel positive regulator of cGAS-STING signaling in SLE pathogenesis. Our results demonstrate that TRIM21 overexpression stabilizes STING by suppressing autophagic degradation, whereas TRIM21 depletion accelerates this clearance process. Mechanistically, TRIM21 catalyzes the K63-linked polyubiquitylation of the selective autophagy receptor p62/SQSTM1, disrupting its interaction with STING. This post-translational modification prevents the sequestration of STING into autophagosomes, thereby stabilizing the adaptor protein and amplifying downstream type I interferon responses. Our findings reveal a previously unrecognized regulatory circuit in which TRIM21 orchestrates cross-talk between ubiquitin signaling and autophagy to control STING turnover. The TRIM21-p62 axis represents a potential therapeutic target for attenuating pathological interferon production in STING-dependent autoimmune disorders. This work advances our understanding of immune regulation by demonstrating how E3 ligase-mediated ubiquitin modifications modulate cargo recognition in selective autophagy pathways. The identified mechanism provides new insights into the molecular interplay between protein ubiquitylation and autophagic degradation in maintaining the innate immune balance, offering novel perspectives for developing targeted therapies against interferonopathies associated with cGAS-STING hyperactivity.

## Introduction

Systemic lupus erythematosus (SLE) is a complex multisystem autoimmune disorder characterized by aberrant immune activation, leading to widespread inflammation and heterogeneous clinical presentations [1]. Contemporary research has highlighted the pivotal role of type I interferon (IFN-I) dysregulation in SLE pathogenesis, where sustained IFN signaling perpetuates immune activation and maintains autoimmune processes [2]. Notably, the cGAS-STING signaling axis has emerged as a critical mediator of type I IFN production and proinflammatory cytokine secretion in innate immune responses [ 3– 5] . The molecular cascade initiates when cytosolic double-stranded DNA (dsDNA) activates cyclic GMP-AMP synthase (cGAS), which catalyzes the synthesis of the second messenger 2′3′-cGAMP [ 6, 7] . This cyclic dinucleotide subsequently binds to and activates stimulator of interferon genes (STING), triggering its translocation from the endoplasmic reticulum to the Golgi apparatus. At this site, STING recruits and phosphorylates TANK-binding kinase 1 (TBK1), which in turn activates interferon regulatory factor 3 (IRF3) through phosphorylation. The nuclear translocation of activated IRF3 ultimately drives the transcription of type I interferons and interferon-stimulated genes (ISGs) [ 6, 8] . Mounting evidence suggests that STING, a central adaptor protein in the cGAS-STING signaling pathway, is involved in the pathogenesis of autoinflammatory and autoimmune disorders [ 9, 10] , such as systemic lupus erythematosus, Aicardi-Goutières syndrome (AGS), and STING-associated vasculopathy with onset in infancy (SAVI) [ 11– 13] . This growing body of evidence underscores the critical need for precise regulation of STING activity to maintain immunological equilibrium and prevent autoimmune dysregulation. Importantly, while the involvement of STING in disease pathogenesis is well documented, the comprehensive regulatory network controlling its activation, trafficking, and downstream signaling remains incompletely characterized.

Autophagy serves as an essential cellular quality control mechanism that maintains homeostasis through the encapsulation of damaged organelles, misfolded proteins, and invading pathogens within double-membraned autophagosomes for subsequent lysosomal degradation. The emerging recognition of autophagy as a precision-targeted degradation system stems from the expanding repertoire of cargo receptors, including p62/SQSTM1, FUNDC1, OPTN, NDP52, and NBR1, which are responsible for recognizing cargoes and facilitating the attachment of ATG8 family proteins to ubiquitin chains or other selective signals on cargoes [14]. Previous research has indicated that STING not only plays a crucial role in activating interferon production but also directly participates in the autophagy process. Through interacting with the autophagy-related protein LC3, STING promotes the formation of autophagosomes and autophagy degradation. Simultaneously, activated STING facilitates its own degradation via autophagy [ 15, 16] . Recent studies have highlighted the importance of autophagy in regulating STING level and activity, particularly through the action of the autophagy receptor p62/SQSTM1; p62/SQSTM1 is known to mediate the selective autophagic degradation of various substrates, including STING. Under conditions of cellular stress or infection, p62/SQSTM1 can bind to STING, facilitating its delivery to autophagosomes for degradation. This process is essential for maintaining cellular homeostasis and preventing excessive inflammatory responses [17]. Furthermore, past studies have demonstrated that p62/SQSTM1 is able to bind to ubiquitinated proteins, thereby promoting autophagy degradation by these proteins. Moreover, the ubiquitination status of p62/SQSTM1 affects its interaction with other proteins, which in turn regulates cellular physiological and pathological processes [ 18, 19] . Both autophagy and the ubiquitin‒proteasome system can regulate cGAS-STING signaling, but the specific regulatory mechanisms governing the interplay between STING and autophagy remain poorly understood. Therefore, further investigations are warranted to elucidate the crosstalk between ubiquitination and autophagy.

TRIM21, also referred to as RNF81, Ro52, Ro/SSA, and SSA1, functions as a transcriptional regulator of type I interferon responses and is implicated in the pathogenesis of autoimmune disorders such as systemic lupus erythematosus and Sjögren syndrome. As a member of the TRIM protein family, TRIM21 possesses a molecular architecture consisting of a RING motif, a B-box motif, a coiled-coil domain, and a PRYSPRY region located at the C-terminal end. This structural composition endows TRIM21 with E3 ligase activity, enabling its involvement in the ubiquitination process [20]. Autophagy has been demonstrated to be regulated by ubiquitination mediated by various TRIM proteins [ 21, 22] , with mechanisms encompassing the modulation of autophagy-related signaling pathways, the regulation of core autophagy molecules, and the function of autophagy receptors. TRIM21 has been implicated in a range of biological pathways through its involvement in ubiquitination, including immunity, viral infections, and tumor development [ 23, 24] . However, the extent of the involvement of TRIM21 in these biological pathways remains unclear.

In this study, TRIM21 was shown to play a critical role in positively regulating STING-mediated type I IFN signaling by inhibiting the autophagic degradation of STING via p62/SQSTM1. Specifically, TRIM21 prevents the interaction between STING and p62/SQSTM1 via TRIM21-mediated K63-linked ubiquitination of p62/SQSTM1, which attenuates the binding of p62/SQSTM1 to STING. Collectively, our results offer novel perspectives on the interplay between autophagy and cGAS-STING signaling, highlighting the essential function of TRIM21 in regulating the activity of cGAS-STING signaling.

## Materials and Methods

### Patients and controls

A cohort of 16 female patients meeting the 1997 revised American College of Rheumatology (ACR) classification criteria for systemic lupus erythematosus (SLE) were prospectively enrolled from the Rheumatology and Clinical Immunology Department of the Second Affiliated Hospital, Kunming Medical University (ethical approval No. 2023-83). All the SLE patients were newly diagnosed to avoid medication interference, with an SLE Disease Activity Index (SLEDAI) of 11.56 ± 2.83. The average age of the SLE patients was 25.00 ± 4.46 years. Forty age- and sex-matched healthy volunteers, with an average age of 24.77 ± 5.14 years, and 40 females were included as controls. In addition, three bulk RNA-seq datasets for SLE, including GSE72509, GSE49454, and GSE138458, were obtained from the GEO database ( https://www.ncbi.nlm.nih.gov/) as external validation cohorts. These datasets also include corresponding clinical data from 99 patients and 18 healthy controls, 157 patients and 20 healthy controls, and 312 patients and 24 healthy controls, respectively.

### Isolation of PBMCs from SLE patients and controls

PBMCs were isolated from fresh blood samples collected from 16 patients with SLE and 40 healthy controls using lymphocyte separation medium (P8610; Solarbio, Beijing, China) following the manufacturer’s instructions. The PBMCs were subsequently cultured in RPMI-1640 medium (Gibco, Carlsbad, USA) supplemented with 10% fetal bovine serum (FBS; Gibco) at 37°C in a 5% CO _2_ environment.

### Cell culture and treatment

HEK293T (STCC10301P) cell line was procured from Servicebio (Shanghai, China). HEK293T cells were cultured in Dulbecco’s modified Eagle’s medium (DMEM; Gibco) supplemented with 10% FBS at 37°C in a 5% CO _2_ environment. Similarly, PBMCs were cultured in RPMI 1640 supplemented with 10% FBS at 37°C in a 5% CO _2_ environment. For drug treatment, cells were seeded into 12-well plates at 30%–40% density 24 h before treatment. The dimethyl sulfoxide (DMSO) stocks of the agents used, including MG132 (HY-13259; MCE, Monmouth Junction, USA), chloroquine (HY-17589A; MCE), were diluted to appropriate concentrations with the cell culture medium. The same volume of 1% DMSO was used as the control treatment. Cells were transfected with HT-DNA (D6898; Sigma, St Louis, USA) at a concentration of 2 μg/mL using the Lipofectamine 3000 reagent (L3000001; Invitrogen, Carlsbad, USA). At 24 h after transfection, cells were harvested for further study.

### Real-time quantitative polymerase chain reaction (qRT-PCR)

Total RNA was extracted using TRNzol reagent (DP424; TIANGEN, Beijing, China), followed by cDNA synthesis using the iScript cDNA Synthesis kit (1708890; Bio-Rad, Hercules, USA), with all reagents employed in accordance with the manufacturer’s guidelines. Amplification was conducted in an ABI 7500 real-time PCR system (Applied Biosystems, Foster City, USA), and the sequences of primers used were as follows: *IFN-β*, forward primer: 5′-CTCCACTACAGCTCTTTCCATGA-3′, reverse primer: 5′-ATTCAAGCCTCCCATTCAATTGC-3′; *STING1*, forward primer: 5′-CATGGGCTGGCATGGTCATATTA-3′, reverse primer: 5′-GCAGTTTATCCAGGAAGCGAATG-3′; and *β-actin*, forward primer: 5′-CCTTCCTGGGCATGGAGTC-3′, reverse primer: 5′-TGATCTTCATTGTGCTGGGTG-3′.

### Western blot analysis and immunoprecipitation assay

Cells were lysed in RIPA lysis buffer (P0013B; Beyotime, Shanghai, China) supplemented with a protease inhibitor and phosphatase inhibitor cocktail (P1045; Beyotime). The lysates were then centrifuged at 15,000 *g* for 15 min, and the resulting supernatants were collected for protein concentration measurement using a BCA assay kit (P0012; Beyotime). The protein samples were subsequently separated by 12% SDS-PAGE, transferred onto PVDF membranes (Millipore, Billerica, USA), and blocked with non-fat dried milk at room temperature for 2 h. The membranes were then incubated overnight at 4°C with the following primary antibodies: anti-TRIM21 antibody (92043S; CST, Danvers, USA), anti-STING antibody (13647; CST), anti-p62 antibody (88588S; CST) and anti-β-actin antibody (66009-1-Ig; Proteintech, Wuhan, China), followed by incubation with HRP-conjugated goat anti-rabbit (H + L) or goat anti-mouse (H + L) secondary antibody (GB23303 or GB23301; Servicebio) for 1 h at room temperature. The protein bands were visualized using a chemiluminescent substrate (P0018S; Beyotime). Owing to the similar molecular weights of TRIM21, STING and β-actin, they are shown together on the same blot. ImageJ was used to perform densitometric analysis.

For immunoprecipitation, cells were washed with ice-cold PBS containing protease inhibitors and lysed as described above. The 2 mg cell lysate was incubated with 2 μg primary antibodies and protein A/G magnetic beads (HY-K0202; MCE) overnight at 4°C. Immunoprecipitates were washed 4 times with lysis buffer and collected using magnetic stand, and boiled for 10 min. Precipitated proteins were analyzed by western blot analysis using corresponding antibodies. All experiments included negative controls without antibodies to confirm specificity.

### ELISA

PBMCs were either stimulated with a final concentration of 200 pg/mL recombinant IFN-β (10704-HNAS; SinoBiological, Beijing, China) or left unstimulated. The levels of IFN-β in the supernatants were measured using ELISA kit (CSB-E09889h; Cusabio, Wuhan, China). PBMC-derived IFN-β levels were determined by subtracting the levels of IFN-β in unstimulated cultures from those in cultures stimulated with recombinant IFN-β.

### Immunofluorescence staining

Following treatment, the cells were washed with PBS and fixed with 4% paraformaldehyde for 25 min at room temperature and subsequently washed with PBS. The primary antibody, microtubule-associated protein light chain 3 (LC3) (L7543; Sigma) at a dilution of 1:500, was then applied to the cells, which were subsequently incubated at 4°C overnight. After being washed three times with PBS, the cells were exposed to an anti-rabbit Alexa Fluor 488 dye-conjugated antibody (GB25303; Servicebio) at a dilution of 1:1000 for 1 h at room temperature in the dark. The cells were subsequently washed and stained with DAPI (AR1176; Boster, Beijing, China). An anti-fluorescence quencher was subsequently used to seal the slides, after which the cells were analyzed via a confocal fluorescence microscope (FV3000; Olympus, Tokyo, Japan).

### 2′3′-cGAMP quantification

PBMCs were cultured in six-well plates and exposed to HT-DNA (D6898; Sigma) or left untreated. Following treatment, the cells were washed with phosphate-buffered saline (PBS) and lysed with methanol. The lysed cells were then transferred to a 1.5-mL centrifuge tube and allowed to incubate at –20°C for 30 min. Subsequently, the sample was centrifuged at 4°C and 15,000 *g* for 20 min, and the resulting supernatant was collected and dried in a vacuum desiccator. Commercial 2′3′-cGAMP (tlrl-nacga23; Invitrogen) was prepared as a standard sample by diluting it with ammonium acetate to a final concentration of 10 mM. The dried sample was then reconstituted with the same concentration of ammonium acetate and centrifuged at 4°C for 10 min at 15,000 *g*, and the resulting supernatant was collected for quantitative analysis via an LC-MS/MS system which includes an ExionLC coupled with a QTRAP 5500 triple-quadrupole mass spectrometer (AB Sciex, Framingham, USA). An electrospray ion source was utilized in conjunction with positive ionization mode. During the assay, 2 transitions of each target ion (675/524, parent ion/daughter ion) were monitored for the quantification of 2′3′-cGAMP, with a retention time of 1.33 min.

### Plasmid construction and transfection

The expression plasmids used included pCDH-cGAS-GFP + Puro (HG-GHO00900), pCDH-IRF3-GFP + Puro (HG-GHO00903), pCDH-SQSTM1-3xFlag-GFP + Puro (HG-GHO003900), pCDH-STING1-3xMyc-GFP + Puro (HG-GHO00902), and pcDNA3.1-TRIM21-3xFlag-HA (HG-GHO00901). The short hairpin RNA (shRNA) constructs against TRIM21 and SQSTM1 were generated using the pHG-LVsh cloning vector. The target sequence of TRIM21 was 5′-GCGCTTTCTGCTCAAGAATCTCTCGAGAGATTCTTGAGCAGAAAGCGCTTTTT-3′, the target sequence of p62/SQSTM1 was 5′-CCGGGCAACAGCCGTATAGAGAATCTCGAGATTCTCTATACGGCTGTTGCTTTTTG-3′, and the target sequence of shRNA NC was 5′-TTCTCCGAACGTGTCACGT-3′, which were all purchased from HonorGene Technology (Changsha, China). The HA-Ub (176462), Flag-p62 (204576), HA-Ub (K48) (17604), and HA-Ub (K63) (17606) plasmids were purchased from Addgene (Cambridge, USA). Lentiviruses were constructed using the aforementioned plasmids as required, and then transduced into HEK293T cells with polybrene (6 μg/mL; HY-112735; MCE) to establish stable cell lines. For transient transfection, the plasmids were introduced into the cells using Lipofectamine 3000 (Invitrogen) according to the manufacturer’s protocol, and the cells were subsequently cultured for 48 h.

### Cell electroporation

Freshly isolated PBMCs were transfected using the Lonza 4D-Nucleofector® X Unit (Lonza, Basel, Switzerland). Cells were harvested and resuspended in pre-warmed complete RPMI-1640 medium. For each transfection, 1 × 10 ^6^ cells were centrifuged at 300 *g* for 5 min, washed once with PBS, and resuspended in 100 μL of P3 Primary Cell 4D-Nucleofector X kit (V4XP-3024; Lonza) according to the manufacturer’s protocol. The cell suspension was mixed with 2 μg plasmid DNA and electroporated using the pre-optimized program EO-115. After nucleofection, the cells were immediately transferred to a 6-well culture plate containing 2 mL of pre-warmed complete medium, and incubated at 37°C under 5% CO _2_. Half of the cell culture medium was replaced by fresh complete medium every 48 h.

### Ubiquitination assay

The target plasmid, HA-Ub plasmid, and plasmids to be detected for modifying effects were transfected into HEK293T cells. Forty-eight hours later, the supernatant was aspirated, and the cells were lysed by adding 120 μL of SDS lysis buffer (50 mM Tris-HCl, pH 6.8, and 1.5% SDS). The cell lysate was transferred to a 1.5-mL EP tube, denatured at 98°C for 15 min, with gentle mixing every 5 min.

The Flag-p62 beads (HY-K0207; MCE) were placed on ice for 20 min, were gently mixed, and approximately 30 μL of beads was used for each sample. According to the actual amount of beads used, a certain amount of beads was added to a 1.5-mL EP tube, and 1 mL of BSA buffer (50 mM Tris-HCl, pH 6.8, 180 mM NaCl, 0.5% NP-40, and 0.5% BSA) was added and mixed well. The mixture was then centrifuged at 5000 *g* for 2 min and washed three times with BSA buffer.

Thirty microlitres of the denatured protein sample was used as the input. The remaining protein sample was added to the BSA buffer at a ratio of 1:10, gently mixed, added to the pre-treated beads, and incubated at 4°C overnight. The incubated samples were centrifuged at 4°C and 1000 *g* for 5 min. The supernatant was aspirated, and 1 mL of BSA buffer was added and mixed gently. The mixture was centrifuged at 4°C and 1000 *g* for 5 min, and the beads were washed 3 times with Votex. The supernatant was aspirated as much as possible, 25 μL loading buffer (P1040; Solarbio) was added, and the mixture was denatured at 98°C for 10 min to fully detach the proteins from the beads. The supernatant was collected after centrifugation and subject to 6% SDS-PAGE.

### Statistical analysis

SPSS 27.0 software was used for data analysis. The count data were described by *n* (%). The measurement data were first tested for normality via the Shapiro-Wilk test. The measurement indicators that follow a normal distribution or approximate normal distribution are described by the mean ± SD. Fisher’s exact probability method was used to compare the differences in counting indicators between two groups. Independent sample *t* tests were used to compare the differences in normally distributed indicators between the two groups. Mann-Whitney U tests were used to compare the differences in nonnormally distributed indicators between the two groups. Analysis of variance (ANOVA) was used to analyze the differences in normally distributed indicators among multiple groups. S-N-K tests were subsequently used for pairwise comparisons when there was a significant difference among groups. Pearson correlation analysis was conducted to analyze the correlations among the expression levels of the TRIM21, STING, and IFN-β genes. GraphPad Prism 9.0 software was used to visualize the data and draw bar charts and box plots. *P*  < 0.05 was considered statistically significant.

## Results

### TRIM21 is elevated in SLE patients

**Figure 1:**
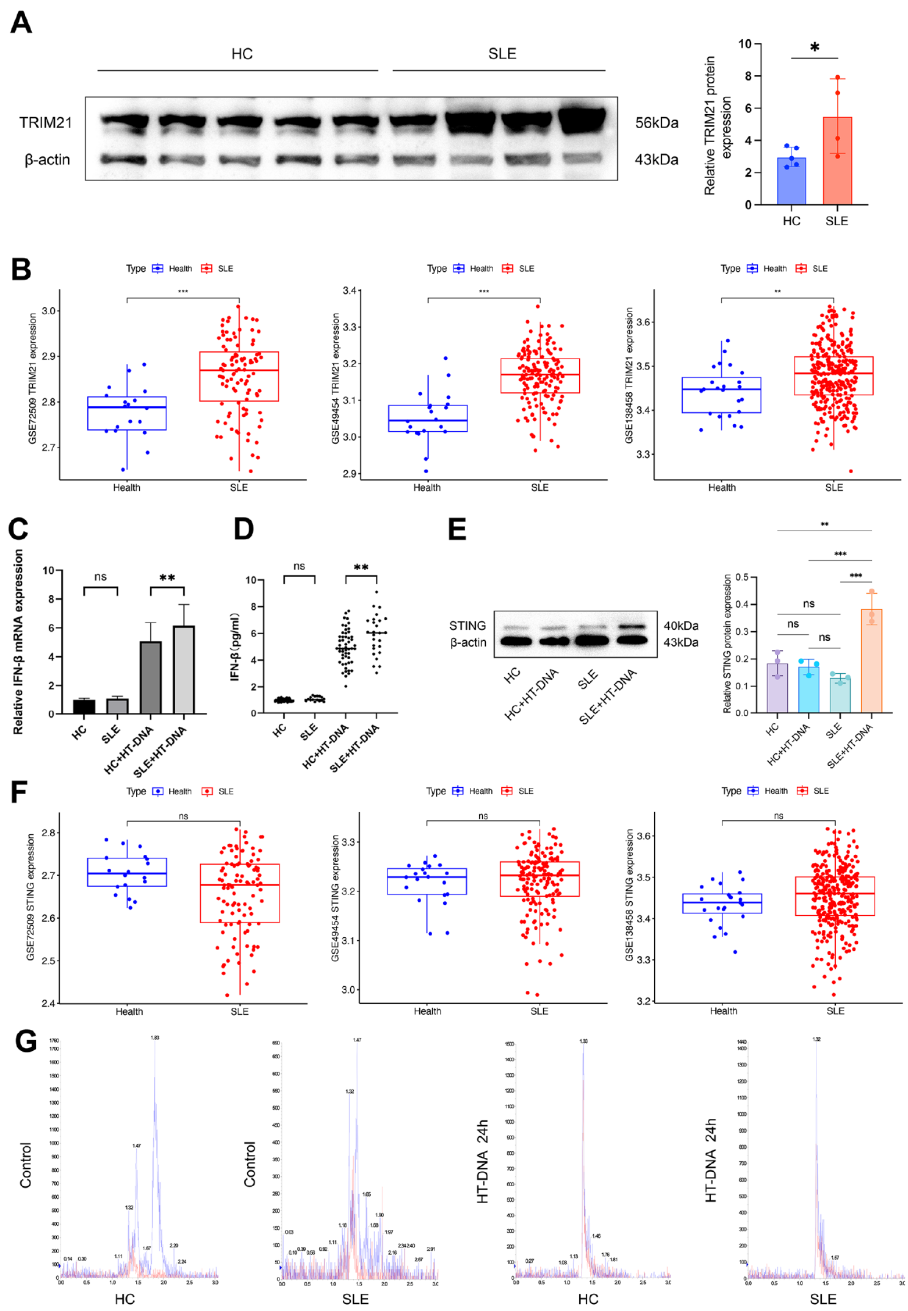
Figure 1 TRIM21 and cGAS-STING signaling activity are elevated in SLE patients A cohort consisting of 40 healthy controls and 16 patients diagnosed with SLE was assembled for the purpose of isolating PBMCs. (A) Western blot analysis of the protein expression level of TRIM21 in PBMCs. (B) TRIM21 mRNA expression in SLE compared with health control in the GSE72509 (99 SLE patients vs 18 health control), GSE49454 (157 SLE patients vs 20 health control) and GSE138458 dataset (307 SLE patients vs 23 health control). (C) The relative mRNA expression levels of IFN-β were measured before and after stimulation with HT-DNA (2 μg/mL) for a duration of 8 h using qPCR. (D) The concentration of IFN-β protein was measured using ELISA both before and after stimulation with HT-DNA at a concentration of 2 μg/mL for a duration of 8 h. (E) Western blot analysis of the protein level of STING in PBMCs isolated from patients (n = 16) and normal individuals (n = 40) before and after transfection with HT-DNA (2 μg/mL) for 8 h. (F) STING mRNA expression in SLE compared with health control in the GSE72509 (99 SLE patients vs 18 health control), GSE49454 (157 SLE patients vs 20 health control) and GSE138458 dataset (307 SLE patients vs 23 health control). (G) In order to quantify the intracellular level of 2′3′-cGAMP in PBMCs, 2′3′-cGAMP was extracted solely from PBMCs following stimulation with or without HT-DNA (2 μg/mL) for a duration of 8 h. Liquid chromatography-tandem mass spectrometry was employed to assess the concentration of 2′3′-cGAMP, with the retention time of the parent/daughter ion (675/524) plotted on the x-axis and the relative intensity of cGAMP’s mass spectrum signal on the y-axis. *P < 0.05, **P < 0.01, and ***P < 0.001, ns, not significant (Two-tailed t-test).

The expression of TRIM21 was analyzed in PBMCs obtained from 40 normal controls and 16 patients with SLE. Consistent with previous studies, western blot analysis revealed significantly elevated expression of TRIM21 in SLE patients compared with that in normal controls ( Figure 1A). This finding was further supported by data from three Gene Expression Omnibus (GEO) datasets, where SLE patients presented significantly higher levels of TRIM21 than healthy controls did ( Figure 1B).

### The cGAS-STING signaling pathway is abnormally activated in SLE patients

To assess the activity of cGAS-STING signaling in patients with SLE, PBMCs were isolated from both healthy controls and individuals with SLE. Since previous studies have shown that cGAS expression levels are comparable between SLE patients and healthy individuals, we performed quantitative analysis of IFN-β and STING to assess the activity of cGAS-STING signaling. On the basis of our research results, we observed that the expressions of IFN-β and STING in the PBMCs of SLE patients were significantly greater after HT-DNA treatment than those of normal individuals ( Figure 1C–E). An additional analysis was conducted to investigate the correlations among the expression levels of TRIM21, STING, and IFN-β. The results revealed a significant positive correlation between TRIM21 and STING expression levels ( *r* = 0.810, *P* = 0.008). Similarly, a significant positive correlation was identified between TRIM21 and IFN-β expression levels ( *r* = 0.828, *P* = 0.006). Moreover, the analysis revealed a strong positive correlation between STING and IFN-β expression levels ( *r* = 0.980, *P*  < 0.001). The data are shown in Table 1. In the datasets GSE72509, GSE49454, and GSE138458, STING expression levels were not different between healthy individuals and SLE patients ( Figure 1F). Our results also revealed that neither IFN-β nor STING was differentially expressed between the normal individuals and the SLE patients before HT-DNA treatment.

**Table:** **Table 1** Correlation analysis among the expression levels of 3 genes

*G*ene expression	*r*/ *P*	*TRIM21*	*STING*	*IFN*- *β*
*TRIM21*	*r*	1.000	0.810	0.828
	*P*	–	0.008	0.006
*STING*	*r*		1	0.980
	*P*		–	< 0.001
*IFN*- *β*	*r*			1.000
	*P*			–

To investigate the potential dysregulation of 2′3′-cGAMP, the canonical second messenger in cGAS-STING signaling, across disease states, we quantitatively profiled the intracellular 2′3′-cGAMP levels in PBMCs via LC-MS/MS. The results revealed no significant difference in 2′3′-cGAMP levels between the two groups treated with or without HT-DNA ( Figure 1G).

We subsequently conducted stratified analyses of clinical-serological profiles in SLE patients categorized by post-stimulation STING activation status (HT-DNA-treated PBMCs) to delineate pathophysiological correlations between disease activity markers and STING responsiveness. The levels of HT-DNA-induced STING were positively correlated with disease activity in SLE patients ( *P* = 0.009). IFN-β expression was significantly higher in STING-positive SLE patients than in STING-negative patients ( *P* = 0.013). Notably, no intergroup disparities were detected in age or anti-TRIM21, anti-dsDNA, or anti-Sm antibody levels ( Table 2). These findings indicate that cGAS-STING signaling is upregulated in patients with SLE, particularly in association with elevated levels of STING.

**Table:** **Table 2** The cGAS-STING signaling activity in PBMCs of SLE patients is associated with clinical characteristics

Variables	Classification	STING (–) group ( *n* = 6)	STING (+) group ( *n* = 10)	Statistics	*P*
Age ( *y*ears)		25.83 ± 4.40	24.50 ± 4.65	0.566	0.580
SLEDAI *s*core		9.33 ± 1.97	12.90 ± 2.42	–3.041	0.009
IFN-β (pg/mL)		5.71 ± 0.94	7.13 ± 0.97	–2.857	0.013
Anti-TRIM21	Negative	1 (16.7)	2 (20.0)		1.000
	Positive	5 (83.3)	8 (80.0)		
Anti-dsDNA	Negative	5 (83.3)	3 (30.0)		0.119
	Positive	1 (16.7)	7 (70.0)		
Anti-Sm	Negative	4 (66.7)	7 (70.0)		1.000
	Positive	2 (33.3)	3 (30.0)		

SLE, systemic lupus erythematosus; PBMCs, peripheral blood mononuclear cells; STING, stimulator of interferon genes; SLEDAI, SLE Disease Activity Index; IFN-β, interferon beta; anti-TRIM21, anti-tripartite motif 21; anti-dsDNA, anti-double-stranded DNA, anti-Sm, anti-Smith.

### TRIM21 functions as a facilitator of cGAS-STING signaling

**Figure 2:**
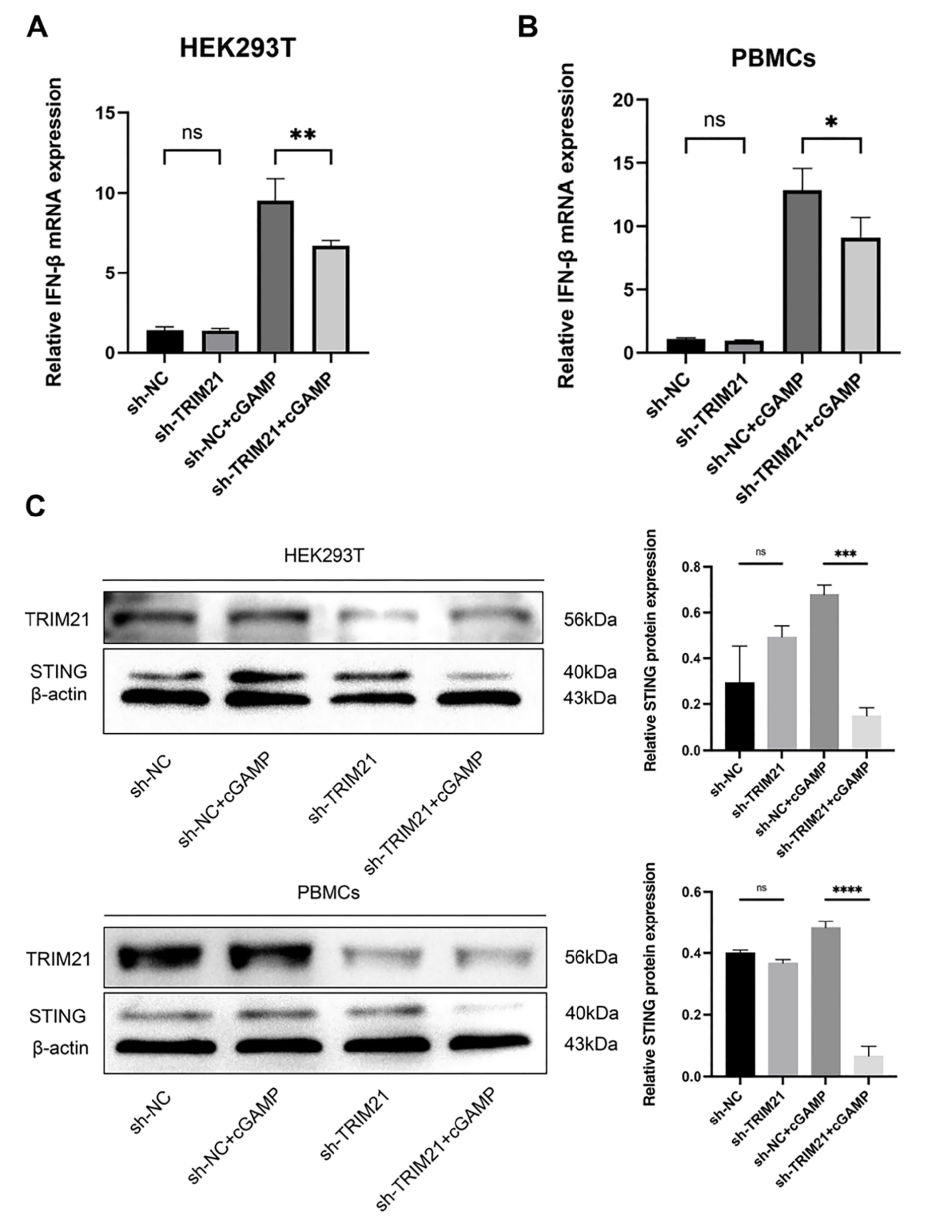
Figure 2 TRIM21 enhances the activity of the cGAS-STING signaling pathway (A) qPCR assays were conducted on HEK293T cells transfected with either a negative control (NC) or sh-TRIM21, followed by stimulation with 2′3′-cGAMP (2 μg/mL) for a duration of 4 h. (B) PBMCs obtained from patients with SLE were transfected with either a negative control (NC) or sh-TRIM21, followed by stimulation with 2′3′-cGAMP (2 μg/mL) for 4 h. Subsequently, the expression of IFN-β was quantified using qPCR. (C) HEK293T cells and PBMCs of SLE patients were transfected with NC or TRIM21 sh-RNAs and subsequently stimulated with or without 2′3′-cGAMP (2 μg/mL) for 4 h. Following immunoprecipitation with anti-TRIM21 or anti-STING antibodies, the lysates were subjected to immunoblotting with the specified antibodies. This study encompasses a minimum of three distinct experiments, with data presented as the mean ± SD. *P < 0.05, **P < 0.01, and ***P < 0.001, ns, not significant, as determined by Student’s t test.

To investigate the potential involvement of TRIM21 in the regulation of cGAS-STING signaling, we utilized short hairpin RNA (shRNA) delivered by lentiviruses to knock down *TRIM21* in HEK293T cells. The shRNAs effectively reduced the levels of TRIM21 in these cells. Subsequent real-time PCR and western blot analyses were performed to confirm changes in the expressions of IFN-β and STING following stimulation with 2′3′-cGAMP. Our results revealed that *TRIM21* knockdown significantly reduced the 2′3′-cGAMP-induced expressions of IFN-β ( Figure 2A) and STING ( Figure 2C) compared with those in control cells. Similarly, shRNA-mediated knockdown of *TRIM21* in PBMCs from both patients with SLE and healthy controls resulted in the same reduction ( Figure 2B). These findings suggest a potential role for TRIM21 in regulating intracellular cGAS-STING signaling.

### TRIM21 modulates cGAS-STING signaling by targeting STING

**Figure 3:**
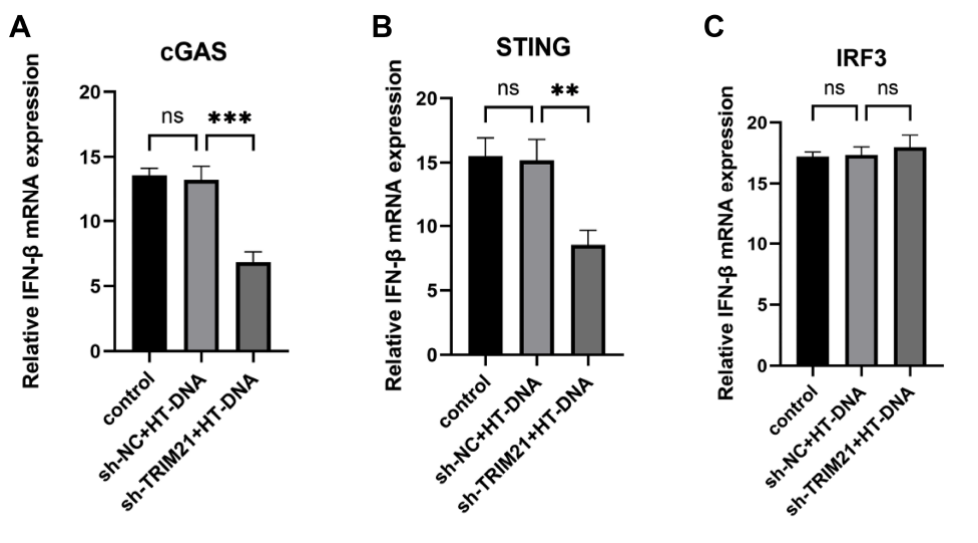
Figure 3 TRIM21 regulates the CGAS-STING1 signaling pathway at the STING level HEK293T cells were transfected with NC or TRIM21 shRNA and cGAS expression plasmids (cGAS), followed by stimulation with HT-DNA (2 μg/mL) for 8 h. The induction of IFN-β was subsequently measured using qPCR. (B) HEK293T cells were transfected with NC or TRIM21 shRNA and STING expression plasmids (STING), followed by stimulation with HT-DNA (2 μg/mL) for 8 h. The induction of IFN-β was subsequently measured using qPCR. (C) HEK293T cells were transfected with negative control (NC) or TRIM21 shRNA and IRF3 expression plasmids (IRF3), followed by stimulation with HT-DNA (2 μg/mL) for 8 h. The induction of IFN-β was subsequently measured using qPCR.

To explore the molecular mechanism underlying the TRIM21-mediated regulation of cGAS-STING signaling, we performed overexpression experiments with cGAS, STING, and IRF3 in HEK293T ^sh-TRIM21^ cells. Our results demonstrated that TRIM21 depletion significantly attenuated the IFN-β expression induced by cGAS or STING overexpression ( Figure 3A,B). However, IRF3-mediated IFN-β activation remained unaffected by *TRIM21* knockdown ( Figure 3C). On the basis of the hierarchical organization of the cGAS-STING signaling cascade, we propose that TRIM21 acts at the level of STING, regulating the activity of the cGAS-STING signaling pathway.

### TRIM21 represses the autophagic degradation of STING

**Figure 4:**
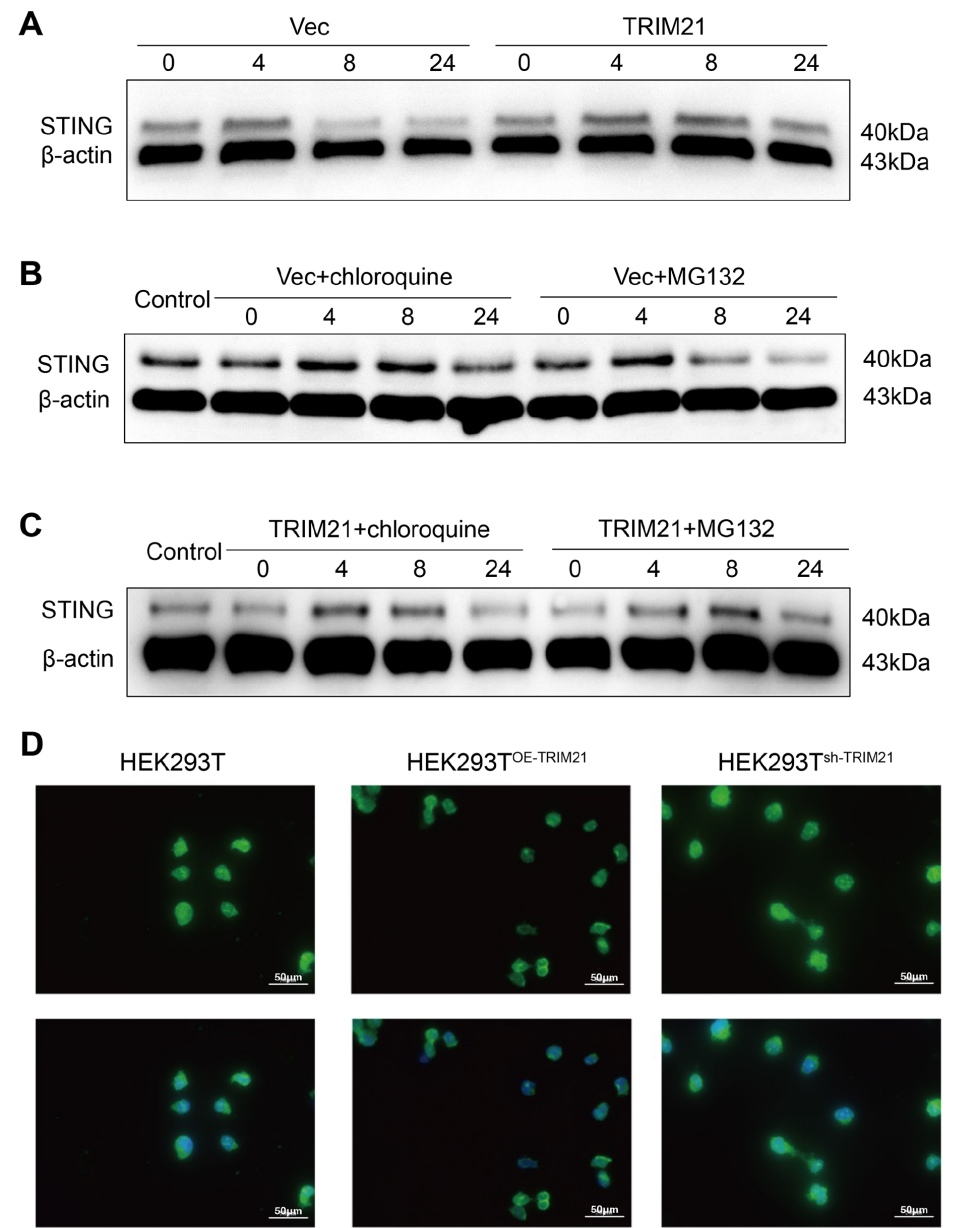
Figure 4 TRIM21 catalyzes K63-linked polyubiquitination of STING to inhibit STING autophagic degradation (A) HEK293T cells were transfected with either control vectors (Vec) or TRIM21 expression plasmids (TRIM21) and subsequently stimulated with 2′3′-cGAMP (2 μg/mL) for 0, 4, 8, or 24 h, and western blot analysis of STING and β-actin expressions were performed on cell lysates. HEK293T cells were transfected with Vec and subsequently stimulated with 2′3′-cGAMP (2 μg/mL) for 0, 4, 8, or 24 h, followed by treatment with MG132 (10 μM) or chloroquine (50 μM). Subsequently, western blot analysis was conducted on cell lysates to assess the expression levels of STING and β-actin. (C) HEK293T cells were transfected with TRIM21 expression plasmids and subsequently stimulated with 2′3′-cGAMP (2 μg/mL) for 0, 4, 8, or 24 h, followed by treatment with MG132 (10 μM) or chloroquine (50 μM). Cell lysates were then collected for western blot analysis of STING and β-actin. (D) Control, OE-TRIM21 and sh-TRIM21 HEK293T cells were stimulated with 2′3′-cGAMP (2 μg/mL) for 8 h and subsequently immunostained with anti-LC3 and DAPI. Scale bar: 50 μm.

Previous studies have indicated that STING undergoes autophagic degradation shortly after activation of the cGAS-STING signaling pathway. In line with these findings, our observations revealed a notable decrease in STING level following treatment with 2′3′-cGAMP. Notably, we found that the overexpression of TRIM21 effectively reversed STING degradation ( Figure 4A). To further elucidate the mechanism by which TRIM21 influences the degradation of STING through autophagy, we conducted experiments using chloroquine, an autolysosome inhibitor, and MG132, a proteasome inhibitor. Our study revealed that chloroquine, rather than the proteasome inhibitor MG132, effectively decreased STING protein level ( Figure 4B). Interestingly, in the TRIM21 overexpression group, there was no significant difference in the impact of chloroquine or MG132 on STING expression ( Figure 4C), indicating that TRIM21 may inhibit STING degradation during autophagy. In support of this hypothesis, ectopic expression of TRIM21 led to a notable decrease in the expression of LC3, whereas knockdown of *TRIM21* resulted in elevated LC3 level in HEK293T cells ( Figure 4D), suggesting that TRIM21 suppresses autophagic activity. Taken together, these findings indicate that TRIM21 inhibits the autophagic degradation of STING, thereby promoting the production of type I interferons via the cGAS-STING signaling pathway.

### TRIM21 suppresses the interaction between p62/SQSTM1 and STING through p62/SQSTM1 ubiquitylation

**Figure 5:**
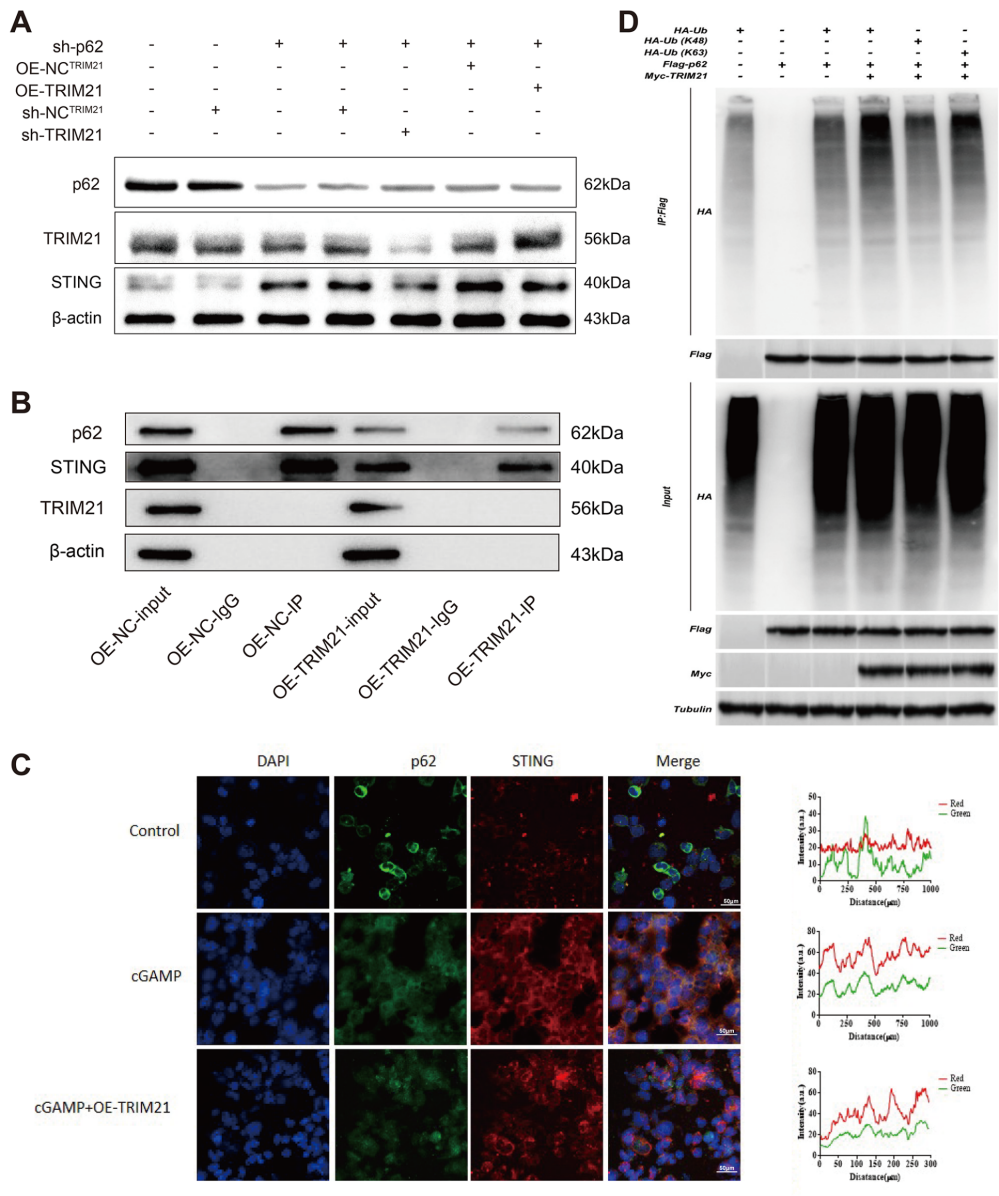
Figure 5 TRIM21 catalyzes K63-linked polyubiquitination of p62/SQSTM1 and suppresses the interaction between p62/SQSTM1 and STING (A) sh-p62 and sh-TRIM21 or OE-TRIM21 were transfected into HEK293T cells, which were subsequently collected and lysed 24 h post-transfection for western blot analysis of p62/SQSTM1, TRIM21, STING, and β-actin. (B) HEK239T cells were transfected with either NC or overexpression TRIM21 (OE-TRIM21) plasmids. Following immunoprecipitation with anti-p62 or normal IgG, the lysates were subjected to immunoblotting using the specified antibodies. (C) HEK293T cells were transfected with NC or OE-TRIM21 plasmids, followed by stimulation with or without 2′3′-cGAMP (2 μg/mL) for 4 h. Subsequently, the cells were fixed, stained with anti-p62 antibody (green) and anti-STING antibody (red), and visualized by confocal microscopy. Scale bar: 50 μm. (D) HEK293T cells were transfected with plasmids encoding Flag-p62, HA-Ub, HA-Ub(K48), HA-Ub(K63), and Myc-TRIM21. Subsequently, after 24 h, the cells were subjected to ubiquitylation assay.

In a previous study, p62/SQSTM1 was shown to interact with STING and promote its degradation through autophagy [15]. To investigate the potential involvement of p62/SQSTM1 in TRIM21-mediated autophagic degradation of STING, cell lines with *p62*/ *SQSTM1* knockdown were generated, revealing an increase in STING expression in HEK293T cells upon p62/SQSTM1 depletion. Furthermore, when *p62*/ *SQSTM1*-knockdown cells were transfected with *TRIM21* knockdown or overexpression plasmids, there were no significant changes in the expression of STING. These findings indicate that the impact of TRIM21 on STING is mediated through p62/SQSTM1 ( Figure 5A). To determine whether TRIM21 affects the recruitment of p62/SQSTM1 to STING, we assessed the interaction between p62/SQSTM1 and STING in HEK293 cells overexpressing TRIM21. Interestingly, the interaction between p62/SQSTM1 and STING was reduced in TRIM21-overexpressing cells compared with those in control cells following stimulation with 2′3′-cGAMP ( Figure 5B,C). In summary, these findings demonstrate that TRIM21 hinders the interaction between p62/SQSTM1 and STING, ultimately leading to the suppression of the autophagic degradation of STING.

As previously stated, TEIM21 is a well-established E3 ubiquitin ligase. To determine whether TRIM21 inhibits p62/SQSTM1 function via ubiquitination, we conducted a co-expression experiment involving HA-ubiquitin, Flag-p62, and Myc-TRIM21 in HEK293T cells, which revealed that Myc-TRIM21 markedly increased the overall ubiquitination of Flag-p62. Subsequent analysis indicated that, unlike K48-linked ubiquitination, TRIM21 facilitates K63-linked ubiquitination of p62/SQSTM1 ( Figure 5D). These findings suggest that TRIM21 catalyzes the K63-linked polyubiquitination of p62/SQSTM1, thereby impeding p62-mediated autophagic degradation of STING.

## Discussion

cGAS-STING signaling plays a crucial role in host defense against infections by preventing and resisting pathogen invasion. However, dysregulation or excessive activation of this pathway can result in severe autoinflammatory and autoimmune disorders [25]. Recent research has linked cGAS-STING signaling to the development and progression of autoimmune diseases. Additionally, evidence suggests that there is a STING-dependent interaction between cGAS-STING signaling and autophagy [9]. Emerging evidence suggests that STING has dual functions in immune activation and autophagy induction. Specifically, STING activation facilitates the generation of type I interferon and inflammatory cytokines and triggers STING-dependent autophagy, which degrades STING to modulate the immune response and mitigate excessive inflammation-induced tissue damage. This process involves noncanonical autophagy that is not dependent on the ULK1 complex or the Beclin1 complex [16]. STING-induced noncanonical autophagy plays an important role in limiting the inflammatory response and protecting cells from excessive immune responses [ 26, 27] . Recently, the regulatory role of p62/SQSTM1 in cGAS-STING signaling has attracted increasing attention. Not only is p62/SQSTM1 an adapter protein for selective autophagy, but it also plays an important role in various cellular signaling pathways [ 28, 29] . Moreover, p62/SQSTM1 is involved in regulating the degradation of STING. p62/SQSTM1 can recognize and degrade ubiquitinated STING through the selective autophagic pathway, thereby regulating its intracellular level [30]. Another study indicated that the cellular metabolic state may influence the activity of STING [31]. These regulatory mechanisms are particularly important in viral infection and cellular stress responses because hyperactivation of STING may lead to the development of autoimmune diseases. The regulation of STING is characterized by intricate control mechanisms at various levels. A comprehensive understanding of these regulatory processes will not only elucidate the fundamental principles of the immune system but also offer novel insights for the development of therapeutic strategies targeting related diseases.

As a disease characterized by an “interferon signature”, SLE is characterized by elevated level of type I interferon which is linked to pathogenesis and disease activity. Activation of the cGAS-STING signaling pathway is closely related to interferon production. Furthermore, aberrant activation of the cGAS-STING signaling pathway is closely associated with the occurrence of autoimmune diseases, which may be due to immune dysregulation resulted from excessive type I IFN production [32]. Kato *et al*. [5] reported that *STING*-knockout reporter cells displayed reduced ISG-inducing activity when exposed to SLE sera. A study demonstrated that cGAMP level was increased in blood samples from a subset of SLE patients, who also exhibited higher disease activity than those without cGAMP [33]. Furthermore, multiple studies using murine models with lupus-like symptoms have demonstrated the critical role of cGAS-STING signaling in lupus pathogenesis [ 34– 36] . These findings suggest that the cGAS-STING pathway may play a role in the excessive elevation of type I interferon in SLE, potentially contributing to the pathogenesis of the disease. Our research also revealed elevated levels of IFN-β and STING in SLE patients, which correlated with disease activity. However, we did not observe high expression of 2′3′-cGAMP in the PBMCs of SLE patients, nor did we find increased mRNA level of *STING*. These findings suggest possible degradation dysfunction of STING in SLE patients.

**Figure 6:**
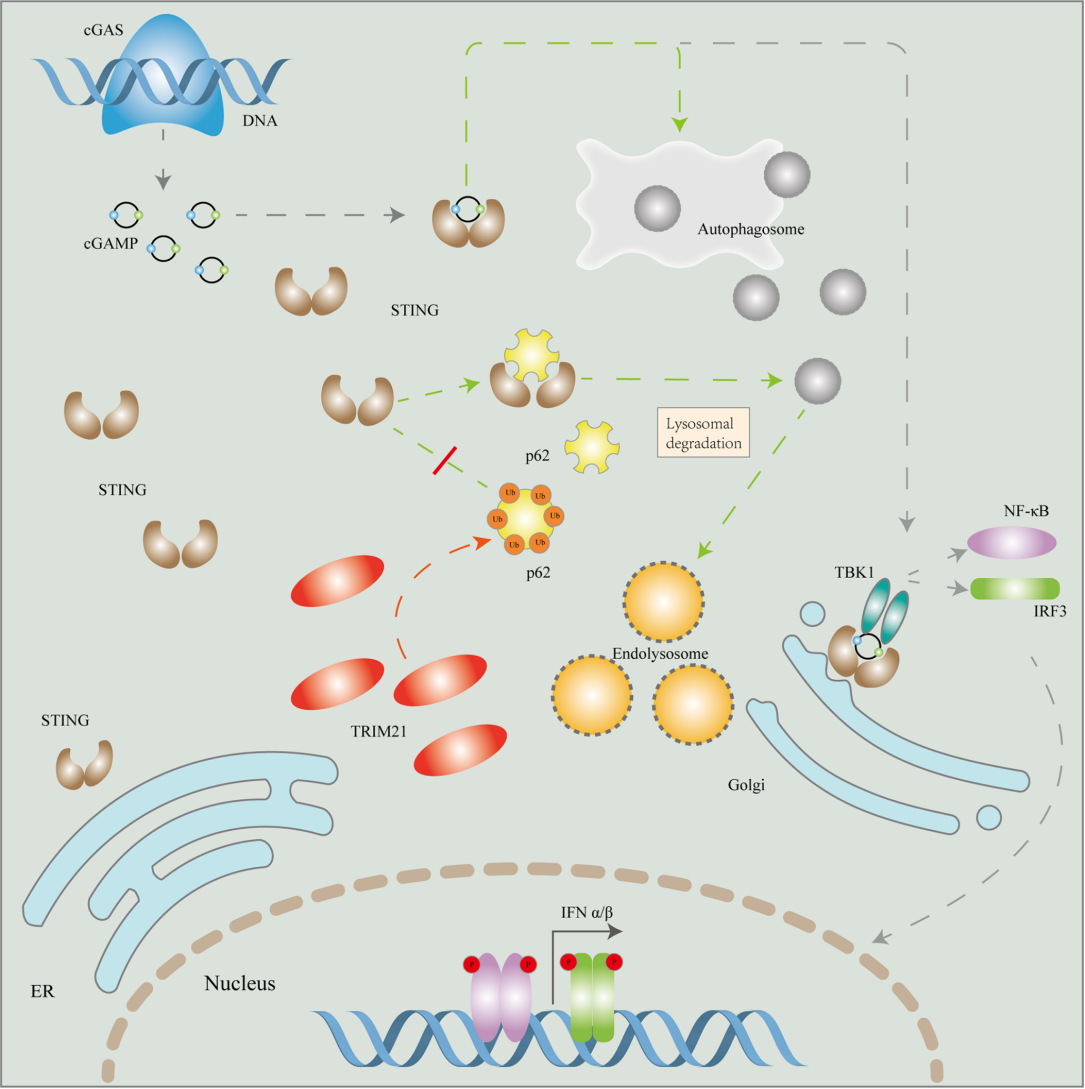
Figure 6 A working model for the positive regulation of the CGAS-STING signaling pathway by TRIM21 TRIM21 catalyzes K63-linked polyubiquitination of the selective autophagy receptor p62, which impairs its dimerization and cargo-sequestration functions, thereby preventing p62-STING interaction. This leads to defective autophagic degradation and cytosolic accumulation of STING, resulting in persistent activation of the cGAS-STING signaling pathway and subsequent aberrant production of type I interferons (IFN-I).

The TRIM family of proteins comprises a diverse array of proteins that possess E3 ubiquitin ligase activity and act as ubiquitin ligases involved in the functional regulation of various cell types, which play significant roles in both physiological and pathological processes *in vivo* [37]. TRIM21 is a member of the TRIM family, and anti-TRIM21 autoantibodies can be detected in approximately 25%–30% of patients with SLE in the clinic [38]. Moreover, research has shown a positive correlation between TRIM21 expression levels in the PBMCs of SLE patients and disease activity [39]. Nevertheless, several studies suggest that TRIM21 functions as a negative regulator of inflammatory responses, indicating that TRIM21 may play a dual role in the immune system, potentially through type I interferon [ 40, 41] . However, the precise mechanism by which TRIM21 contributes to the pathogenesis of SLE remains incompletely understood. Recent studies have shown that TRIM21 inhibits the intracellular dimerization and sequestration of p62/SQSTM1 by modifying the ubiquitination of p62/SQSTM1 [42]. In this study, we identified TRIM21 as a critical positive regulator of STING-mediated type I interferon signaling. By suppressing the autophagic degradation of STING, TRIM21 amplifies cGAS-STING signaling activity, thereby driving excessive type I interferon. Mechanistically, TRIM21 facilitates the K63-linked polyubiquitination of p62/SQSTM1, resulting in hindered transport of STING to lysosomes by inhibiting the specific interaction between p62/SQSTM1 and STING ( Figure 6). Pathologically, TRIM21 expression is upregulated in the PBMCs of patients with SLE. Notably, *TRIM21* silencing attenuated cGAMP-stimulated type I interferon production in these cells.

Our findings indicate that the levels of 2′3′-cGAMP in the PBMCs of SLE patients were not significantly different from those in the PBMCs of normal controls, contrary to the findings of previous studies. Nonetheless, SLE patients demonstrated a notable increase in type I interferon production following stimulation with 2′3′-cGAMP, suggesting a potentially significant role of 2′3′-cGAMP in the pathogenesis of the disease. The variance in the assay outcomes could be attributed to the rapid degradation of 2′3′-cGAMP *in vitro* [43]. This observation underscores the need for further investigation into 2′3′-cGAMP metabolic pathways in the context of SLE. Our study revealed that the cGAS-STING signaling pathway is more frequently activated in PBMCs from patients with SLE following HT-DNA stimulation. This activation may be associated with the inhibition of STING degradation, thereby offering a novel perspective for the diagnosis of SLE. Notably, PBMCs from both SLE patients and healthy controls showed no increase in IFN-β production upon exogenous IFN-β stimulation. This dissociation suggests that IFN-β may not directly drive cGAS-STING pathway activation, further indicating its limited role as a primary effector in SLE pathogenesis. The influence of type I interferon on SLE pathogenesis may be mediated through alternative mechanisms, such as their impact on inflammatory cytokines [44]. Similarly, HT-DNA stimulation of PBMCs from both SLE patients and healthy controls failed to alter intracellular TRIM21 expression levels, suggesting that the elevated TRIM21 observed in SLE patients likely involves additional regulatory mechanisms beyond DNA-sensing pathways.

In summary, our study reveals a novel role of TRIM21 in amplifying cGAS-STING signaling pathway-driven type I interferon production in SLE. Mechanistically, TRIM21 facilitates STING-mediated interferon responses by catalyzing the K63-linked polyubiquitination of p62/SQSTM1, which blocks the autophagic degradation of STING and perpetuates pathological cGAS-STING pathway hyperactivation. These findings establish critical functional crosstalk between ubiquitination and autophagy in SLE pathogenesis. Given the pathogenic role of dysregulated cGAS-STING signaling in autoimmune disorders, targeting TRIM21’s E3 ubiquitin ligase activity could open new avenues for developing precision therapeutics to modulate this pathway in SLE and related conditions.
